# MALT1 positively relates to Th17 cells, inflammation/activity degree, and its decrement along with treatment reflects TNF inhibitor response in ankylosing spondylitis patients

**DOI:** 10.1002/jcla.24472

**Published:** 2022-05-27

**Authors:** Jie Yuan, Lei Xiang, Feng Wang, Lin Zhang, Gaozhan Liu, Xiuli Chang, Anbing Zhang, Ying Tao

**Affiliations:** ^1^ Department of Rheumatology Xiangyang Central Hospital Affiliated Hospital of Hubei University of Arts and Science Xiangyang China

**Keywords:** ankylosing spondylitis, ASAS40 response, MALT1, T helper cells, tumor necrosis factor inhibitor

## Abstract

**Background:**

Mucosa‐associated lymphoid tissue lymphoma translocation protein 1 (MALT1) facilitates CD4^+^ T‐cell differentiation, immune response, inflammation, and osteoclastogenesis. This study aimed to explore the relation between MALT1 and treatment efficacy to tumor necrosis factor inhibitor (TNFi) in ankylosing spondylitis (AS) patients.

**Methods:**

This study recruited 73 AS patients underwent adalimumab treatment. Peripheral blood mononuclear cell (PBMC) was obtained at Week (W) 0, W4, W8, and W12 after treatment initiation; then, MALT1 was measured using RT‐qPCR. Furthermore, PBMC and serum at W0 were proposed to flow cytometry and ELISA for Th1 cells, Th17 cells, IFN‐γ, and IL‐17A levels measurement. Besides, 20 osteoarthritis patients and 20 healthy controls (HCs) were enrolled to detect MALT1.

**Results:**

Mucosa‐associated lymphoid tissue lymphoma translocation protein 1 expression was higher in AS patients compared with HCs (*p* < 0.001) and osteoarthritis patients (*p* < 0.001). Besides, MALT1 expression was positively linked with CRP (*p* = 0.002), BASDAI (*p* = 0.026), PGADA (*p* = 0.040), ASDAS_CRP_ (*p* = 0.028), Th17 cells (*p* = 0.020), and IL‐17A (*p* = 0.017) in AS patients, but did not relate to other clinical features, Th1 cells or IFN‐γ (all *p*>0.050). MALT1 was decreased along with treatment only in AS patients with ASAS40 response (*p* < 0.001), but not in those without ASAS40 response (*p* = 0.064). Notably, MALT1 expression was of no difference at W0 (*p* = 0.328), W4 (*p* = 0.280), and W8 (*p* = 0.080), but lower at W12 (*p* = 0.028) in AS patients with ASAS40 response compared with those without ASAS40 response.

**Conclusion:**

Mucosa‐associated lymphoid tissue lymphoma translocation protein 1 positively correlates with Th17 cells, inflammatory, and activity degree; meanwhile, its decrement along with treatment reflects the response to TNF inhibitor in AS patients.

## Graphical abstract

Mucosa‐associated lymphoid tissue lymphoma translocation protein 1 (MALT1) expression in 73 ankylosing spondylitis (AS) patients was detected at Week (W) 0, W4, W8, and W12 after treatment initiation by RT‐qPCR. Th1 cells, Th17 cells and IFN‐γ, and IL‐17A levels in AS patients were measured at W0 by flow cytometry and ELISA separately. Besides, MALT1 expressions in 20 osteoarthritis patients and 20 healthy controls (HCs) were also detected. MALT1 expression was higher in AS patients compared with HCs and osteoarthritis patients. Besides, MALT1 expression was positively linked with CRP, BASDAI, PGADA, ASDAS_CRP_, Th17 cells, and IL‐17A in AS patients. MALT1 was decreased along with treatment in AS patients, and this trend was more pronounced in those with ASAS40 response. Notably, MALT1 expression was lower at W12 in AS patients with ASAS40 response compared with those without ASAS40 response.

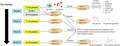

## 1 INTRODUCTION

Ankylosing spondylitis (AS) is a chronic inflammatory disease that predominantly affects the spine, sacroiliac joints, and leads to bone erosion as well as joint deformation; these pathological changes can result in disability and vastly affect the patients’ quality of life.^1^, ^2^ Currently, therapies for AS mainly include physiotherapy, non‐steroidal anti‐inflammatory drugs (NSAIDs), glucocorticoids, and biological agents.^2^, ^3^ Typically, the utilization of TNF inhibitors, including adalimumab, infliximab, and golimumab, has largely improved the management of AS patients.^4^, ^5^ Whereas a certain part of the patients cannot well respond to TNF inhibitor treatment, thus it is of importance to explore more indicators to forecast the treatment efficacy of TNF inhibitors and therefore realize better supervision for AS patients.

Mucosa‐associated lymphoid tissue lymphoma translocation protein 1 (MALT1) is an essential regulator of multiple immune‐related pathways.^6^, ^7^, ^8^ Interestingly, it is illustrated that MALT1 elevates the proportion of Th1 and Th17 cells and activates the T‐cell receptor pathway.^9^, ^10^, ^11^ Additionally, MALT1 is excessively expressed in osteoclasts; meanwhile, inhibiting MALT1 suppresses the differentiation of monocytes into osteoclasts in mice.^12^, ^13^ What is more, a preclinical study in the field of AS discloses that inhibiting MALT1 represses the level of inflammation via regulating NF‐κB signaling.^14^ Considering these biological processes (T‐cell differentiation, the formation of osteoclasts, and inflammation recruitment) play essential roles in the pathogenesis of AS, we further hypothesized that MALT1 could serve as an indicator for AS management. Hence, the present study aimed to explore the relation of MALT1 with clinical features and treatment efficacy to TNF inhibitor in AS patients.

## 2 METHODS

### 2.1 Subjects

This study consecutively recruited 73 AS patients who were going to adopt adalimumab treatment from February 2019 to January 2021. The patients who met the following criteria were eligible for recruitment: (a) diagnosed as AS in accordance with the Assessment of Spondyloarthritis International Society (ASAS) classification criteria^15^; (b) confirmed as active disease, which was defined as Bath Ankylosing Spondylitis Disease Activity Index (BASDAI) >4 and Ankylosing Spondylitis Disease Activity Score based on C‐reactive protein (CRP) (ASDAS_CRP_) >2.1; (c) over 18 years old; (d) about to be treated with adalimumab for at least 12 weeks; and (e) volunteered to provide peripheral blood (PB) samples. The patients with the following conditions were excluded: (a) complicated with active hepatitis; (b) presented with infection; (c) allergic to adalimumab; (d) previously treated with adalimumab but had poor efficacy; and (e) had liver, kidney, heart, or respiration dysfunction. Additionally, during the same period, a total of 20 osteoarthritis (OA) patients, as well as 20 healthy subjects, were included in the study as disease controls and health controls (HCs), respectively. The current study was permitted by Ethics Committee of Xiangyang Central Hospital, Affiliated Hospital of Hubei University of Arts and Science.

### 2.2 Data and samples collection

Clinical characteristics of AS patients were obtained before treatment initiation, including age, gender, disease duration, treatment history, human leukocyte antigen‐B27 (HLA‐B27), biochemistry indexes, and disease activity scores. PB samples of AS patients were collected at initiation (*N* = 73), 4 weeks (W4) (*n* = 71), W8 (*n* = 67), and W12 (*n* = 62) after the initiation of treatment. Besides, PB samples were also collected from OA patients and HCs after inclusion. After sample collection, peripheral blood mononuclear cell (PBMC) samples and serum samples were isolated for determination.

### 2.3 Examination of samples

Peripheral blood samples of 44 AS patients were available to detect T helper 1 (Th1) cell percentage and T Th17 cell percentage by flow cytometry using Human Th1/Th17 Phenotyping Kit (Thermo Fisher). Serum samples of all AS patients were applied to assess the level of interferon‐gamma (IFN‐γ) and interleukin 17A (IL‐17A) by enzyme‐linked immunosorbent assay (ELISA) using Human IFN‐γ ELISA Kit and Human IL‐17A ELISA Kit (Sangon Biotech), respectively. The experimentations were performed based on the manuals of manufacturers. The collected PBMC samples of all subjects (AS patients, OA patients, and HCs) were used to determine MALT1 expression by reverse transcription‐quantitative polymerase chain reaction (RT‐qPCR).

The total RNA was primarily fetched with RNeasy Protect Mini Kit (Qiagen). Besides, complementary DNA was then synthesized using PrimeScript™ RT reagent Kit (Takara). Meanwhile, qPCR was performed using KOD SYBR^®^ qPCR Mix (Toyobo). Moreover, Glyceraldehyde‐3‐phosphate dehydrogenase (GAPDH) was chosen to be the internal reference for MALT1, and the qPCR process was referred to preceding research.^16^ Then, the 2^−ΔΔCt^ method was applied to calculate MALT1 relative expression.

### 2.4 Treatment and assessment

The AS patients were treated with adalimumab (Abbott Laboratories) subcutaneously at a dose of 40 mg every 2 weeks for 12 weeks. During the treatment, the treatment response (ASAS40 response was defined as an improvement of ≥40% and ≥2 units on a scale of 10 in at least three of the four ASAS main domains and no worsening at all in the remaining domain) was evaluated at W2, W4, W8, and W12 in line with ASAS40 response criteria.^15^ During the follow‐up, 11 (15.1%) AS patients dropped out from the study early, including 8 cases due to loss of follow‐up, 2 cases due to lack of treatment efficacy, and 1 case due to withdrawal of informed consent. For these patients, the missing data were processed using the last observation carried forward (LOCF) method.

### 2.5 Statistics

Statistical analyses were completed using SPSS V.24.0 (IBM Corp.), and graphs were plotted using GraphPad Prism V.6.01 (GraphPad Software Inc.). In study analysis, the AS patients were categorized as response AS patients and non‐response AS patients according to the assessment of ASAS40 response at W12. Comparative analysis was completed using the Mann–Whitney U test or chi‐squared test as appropriate. The ability of MALT1 expression in differentiating subjects was evaluated using the receiver operating characteristic (ROC) curve. Correlation analysis was carried out using Spearman's rank correlation test or Mann–Whitney U test. Repeated measures of MALT1 expression were analyzed using the Friedman test. *p* < 0.05 was considered statistically significant.

## 3 RESULTS

### 3.1 Clinical characteristics

The mean age of AS patients was 38.3 ± 8.6 years, with 10 (13.7%) females and 63 (86.3%) males (Table 1). The median disease duration was 5.1 (interquartile range (IQR): 3.3–7.5) years. The median CRP and erythrocyte sedimentation rate (ESR) were 26.4 (IQR: 19.0–36.8) mg/L and 31.7 (IQR: 22.5–42.6) mm/H, respectively. Besides, mean BASDAI, bath ankylosing spondylitis functional index (BASFI), total back pain, patient's global assessment of disease activity (PGADA), and ASDAS_CRP_ were 5.8 ± 1.1, 4.8 ± 1.2, 5.4 ± 1.4, 5.6 ± 1.5, and 3.6 ± 0.9, separately. More detailed information was listed in Table 1.

**TABLE 1:** Clinical characteristics of AS patients

Items	AS patients (*N* = 73)
Age (years), mean ± SD	38.3 ± 8.6
Gender, *n* (%)	
Female	10 (13.7)
Male	63 (86.3)
Disease duration (years), median (IQR)	5.1 (3.3–7.5)
History of NSAID, *n* (%)	72 (98.6)
History of TNF inhibitor, *n* (%)	20 (27.4)
HLA‐B27 positive, *n* (%)	66 (90.4)
CRP (mg/L), median (IQR)	26.4 (19.0–36.8)
ESR (mm/H), median (IQR)	31.7 (22.5–42.6)
BASDAI (0–10 cm VAS), mean ± SD	5.8 ± 1.1
BASFI (0–10 cm VAS), mean ± SD	4.8 ± 1.2
Total back pain (0–10 cm VAS), mean ± SD	5.4 ± 1.4
PGADA (0–10 cm VAS), mean ± SD	5.6 ± 1.5
ASDAS_CRP_, mean ± SD	3.6 ± 0.9

Abbreviations: AS, ankylosing spondylitis; ASDAS_CRP_, ankylosing spondylitis disease activity score based on C‐reactive protein; BASDAI, bath ankylosing spondylitis disease activity index; BASFI, bath ankylosing spondylitis functional index; CRP, C‐reactive protein; ESR, erythrocyte sedimentation rate; HLA, human leukocyte antigen; IQR, interquartile range; NSAID, non‐steroid anti‐inflammatory drugs; PGADA, patient's global assessment of disease activity; SD, standard deviation; TNF, tumor necrosis factor; VAS, visual analog scale.

### 3.2 MALT1 expression

**FIGURE 1:**
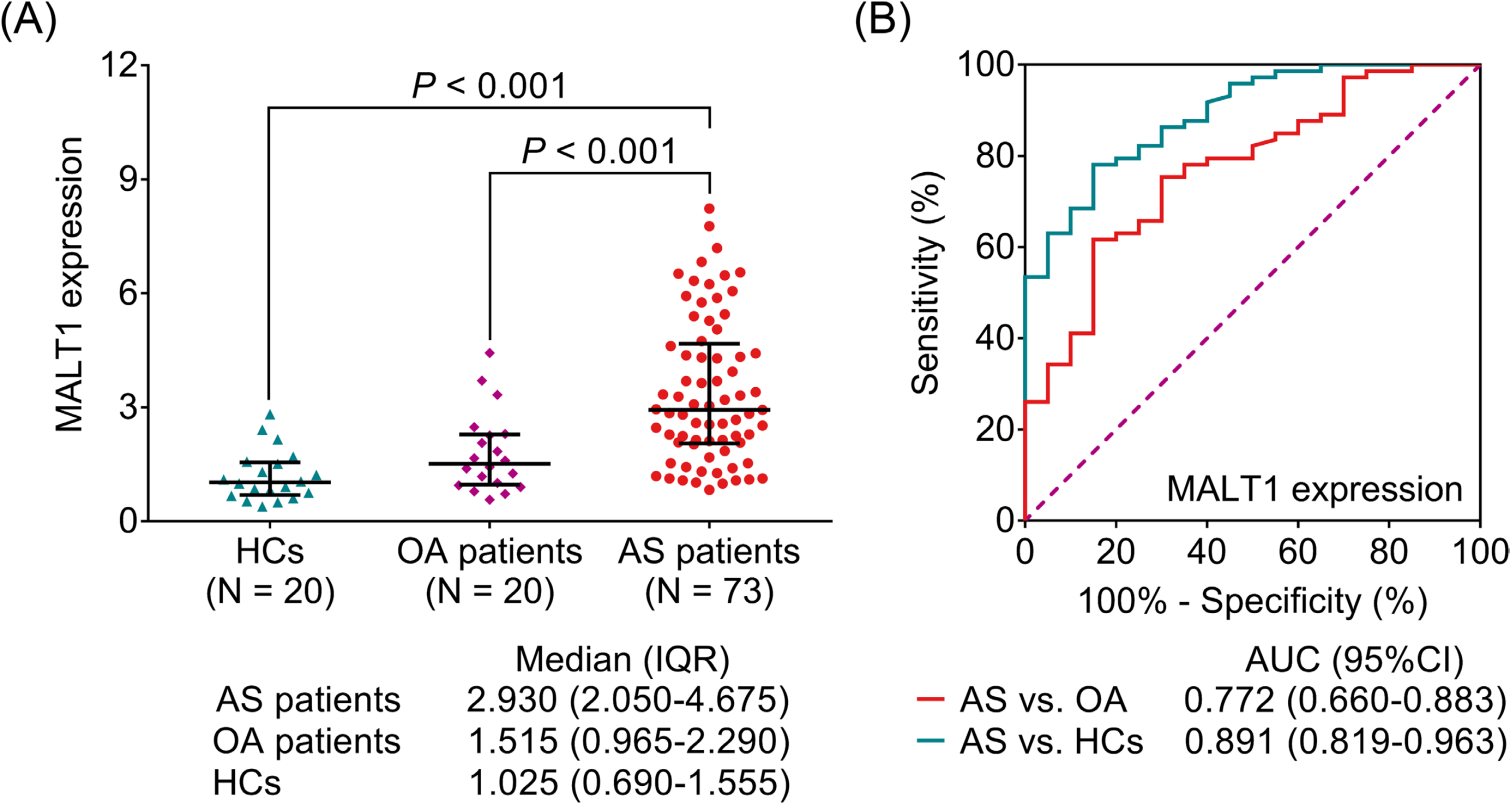
Mucosa‐associated lymphoid tissue lymphoma translocation protein 1 expression was higher in AS patients versus OA patients and HCs. Comparison of MALT1 expression between HCs, OA patients, and AS patients (A); ROC curves of MALT1 expression in distinguishing AS from OA and distinguishing AS from HCs (B)

Mucosa‐associated lymphoid tissue lymphoma translocation protein 1 expression in AS patients (2.930 (IQR: 2.050–4.675)) was higher than that in HCs (1.025 (IQR: 0.690–1.555)) (*p* < 0.001) and OA patients (1.515 (IQR: 0.965–2.290)) (*p* < 0.001) (Figure 1A). Besides, ROC curves were drawn to differentiate the AS patients from HCs and OA patients, which showed that MALT1 expression could discriminate AS patients from HCs (its area under curve (AUC) equaling to 0.891 (95% confidence interval (CI): 0.819–0.963)) and OA patients (its AUC equaling to 0.891 (CI: 0.660–0.883)), respectively (Figure 1B).

### 3.3 Correlation of MALT1 with clinical indexes and Th cells

**FIGURE 2:**
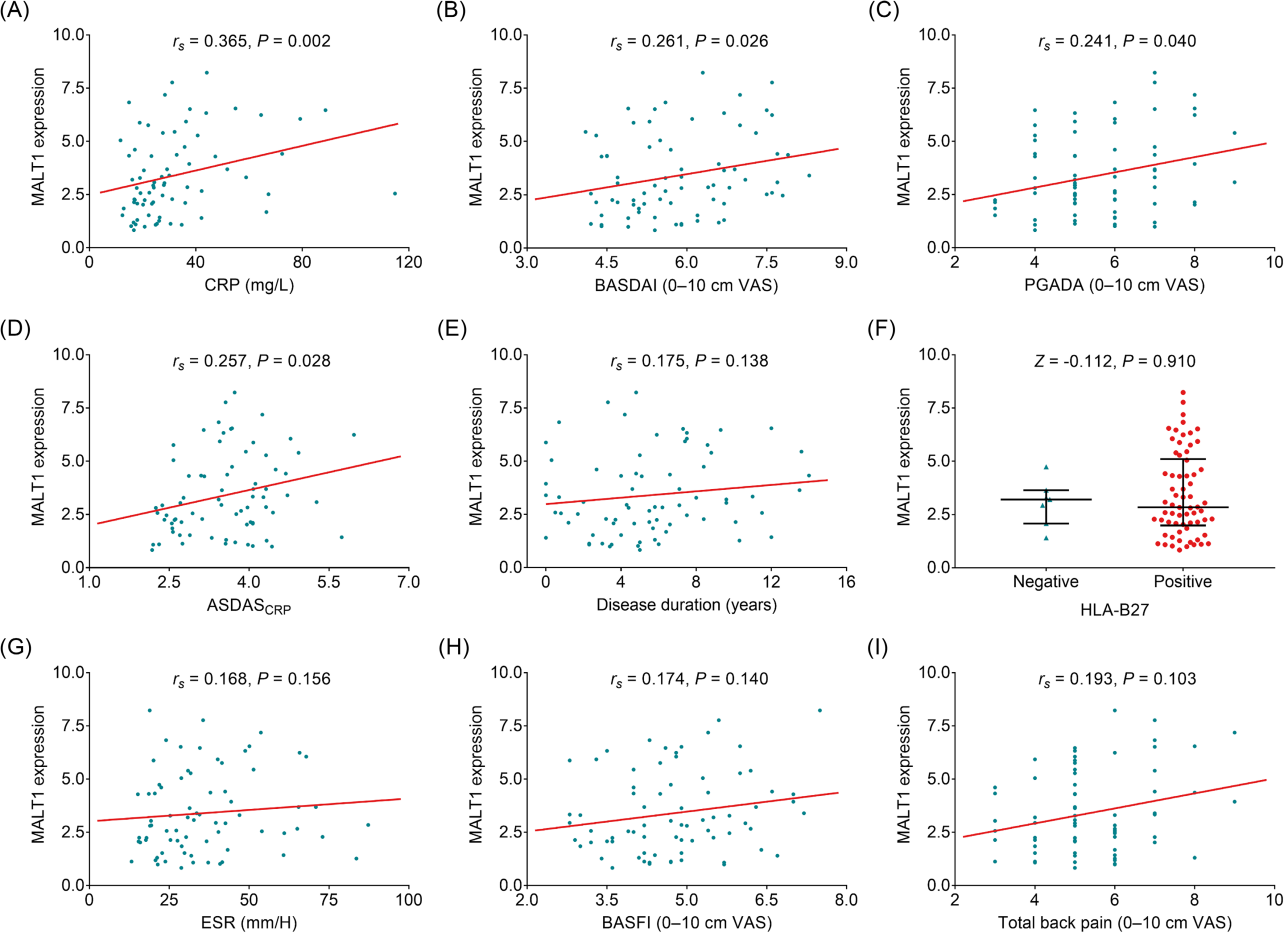
Mucosa‐associated lymphoid tissue lymphoma translocation protein 1 positively correlated with CRP, BASDAI, PGADA, and ASDAS_CRP_. Correlation of MALT1 expression with CRP (A), BASDAI (B), PGADA (C), ASDAS_CRP_ (D), disease duration (E), HLA‐B27 (F), ESR (G), BASFI (H), and total back pain (I) in AS patients

Mucosa‐associated lymphoid tissue lymphoma translocation protein 1 expression was positively linked with CRP (*r*_s_ = 0.365, *p* = 0.002, Figure 2A), BASDAI (*r*_s_ = 0.261, *p* = 0.026, Figure 2B), PGADA (*r*_s_ = 0.241, *p* = 0.040, Figure 2C), and ASDAS_CRP_ (*r*_s_ = 0.257, *p* = 0.028, Figure 2D) in AS patients, whereas MALT1 was not linked with the rest of other clinical indexes, for instance the disease duration, HLA‐B27 positive, ESR, BASFI, or total back pain (Figure 2E–I) in AS patients.

**FIGURE 3:**
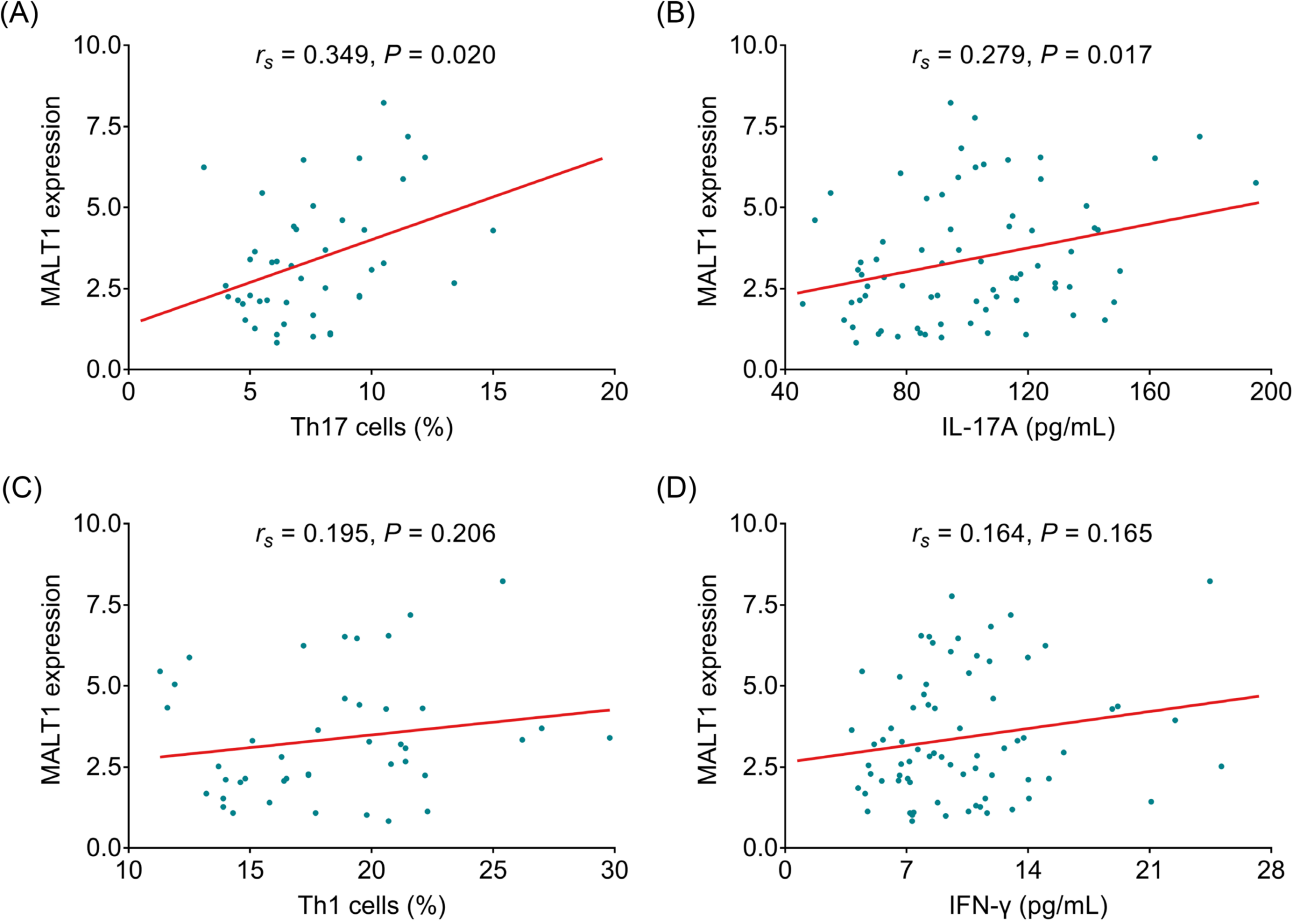
Mucosa‐associated lymphoid tissue lymphoma translocation protein 1 positively correlated with Th17 cells and IL‐17A. Association of MALT1 expression with Th17 cells (A), IL‐17A (B). Th1 cells (C), and IFN‐γ (D) in AS patients

Mucosa‐associated lymphoid tissue lymphoma translocation protein 1 expression was positively correlated with Th17 cells (*r*_s_ = 0.349, *p* = 0.020, Figure 3A) and IL‐17A (*r*_s_ = 0.279, *p* = 0.017, Figure 3B), rather than Th1 cells (*r*_s_ = 0.195, *p* = 0.206, Figure 3C) or IFN‐γ (*r*_s_ = 0.164, *p* = 0.165, Figure 3D) in AS patients. Additionally, the current study also evaluated the correlation of MALT1 with Th1/Th17 ratio in AS patients, which disclosed that MALT1 was not associated with the Th1/Th17 ratio in AS patients (*r*_s_ = −0.250, *p* = 0.102, Figure S1).

### 3.4 MALT1 expression during treatment

**FIGURE 4:**
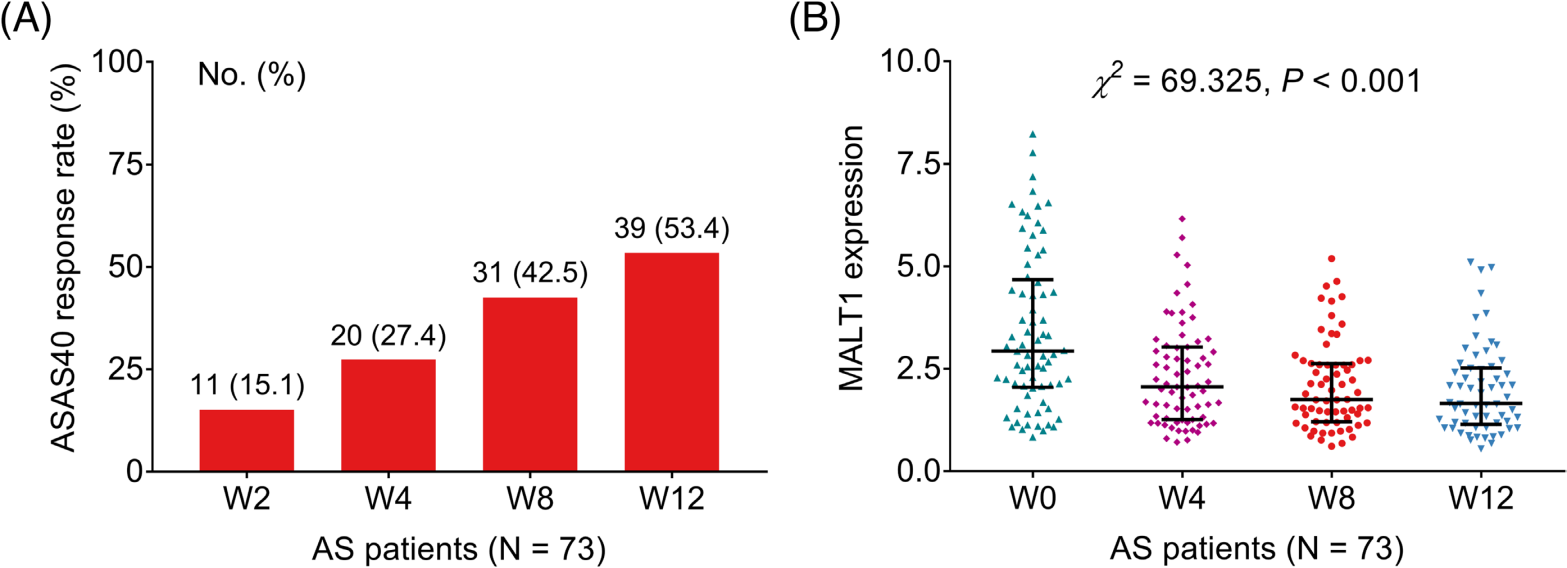
Mucosa‐associated lymphoid tissue lymphoma translocation protein 1 expression decreased during the adalimumab treatment. ASAS40 response rate at W2, W4, W8, and W12 in AS patients (A); MALT1 expression at baseline, W4, W8, and W12 in AS patients (B)

**FIGURE 5:**
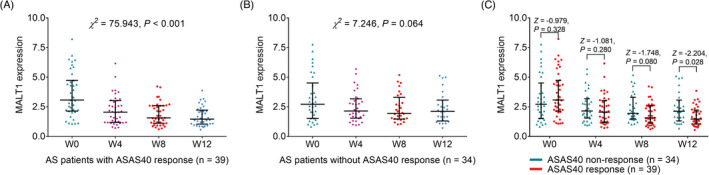
Mucosa‐associated lymphoid tissue lymphoma translocation protein 1 expression decreased in ASAS40 response patients. MALT1 expression in AS patients with ASAS40 response at W0, W4, W8, W12 (A). MALT1 expression in AS patients without ASAS40 response at W0, W4, W8, W12 (B). Comparison of MALT1 expression at W0, W4, W8, W12 between AS patients with ASAS40 response and those without ASAS40 response (C)

ASAS40 response rates at W2, W4, W8, and W12 were 15.1%, 27.4%, 42.5%, and 53.4%, separately (Figure 4A). Besides, MALT1 was decreased gradually in AS patients during the adalimumab treatment (*χ^2^* = 69.325, *p* < 0.001, Figure 4B). In detail, MALT1 was decreased gradually in patients with ASAS40 response (*χ^2^* = 75.943, *p* < 0.001, Figure 5A), whereas this decreasing trend was insignificant in patients without ASAS40 response (*χ^2^* = 7.246, *p* = 0.064, Figure 5B). More importantly, MALT1 expression was lower at W12 (*Z* = −2.204, *p* = 0.028) in patients with ASAS40 response compared with patients without ASAS40 response (Figure 5C), whereas MALT1 expression was of no difference at baseline (*Z* = −0.979, *p* = 0.328), W4 (*Z* = −1.081, *p* = 0.280) or W8 (*Z* = −1.748, *p* = 0.080) between patients with ASAS40 response and patients without ASAS40 response.

Additionally, MALT1 positively correlated with Th17 cells in patients with ASAS40 response (*r*_s_ = 0.421, *p* = 0.041, Table S1); meanwhile, MALT1 positively related to IL‐17A in patients without ASAS40 response (*r*_s_ = 0.389, *p* = 0.023), whereas MALT1 was not associated with other Th cells or cytokines in response patients or non‐response patients (all *p* > 0.05). Then, it was also revealed that a history of TNF inhibitors did not affect the responsiveness of TNF inhibitor in AS patients (*χ* ^2^ = 0.786, *p* = 0.375, Figure S2).

Beyond that, the present study detected the variation of MALT1 expression in AS patients with or without a history of TNF inhibitors, which disclosed that there was no difference in MALT1 expression at W0, W4, W8, and W12 between AS patients with a history of TNF inhibitors and those without a history of TNF inhibitors (all *p* > 0.05, Figure S3).

## 4 DISCUSSION

Mucosa‐associated lymphoid tissue lymphoma translocation protein 1, which is also known as para‐caspase, is reported to be dysregulated in a series of autoimmune diseases: One research finds that MALT1 is abnormally elevated in ulcerative colitis and Crohn's disease patients.^17^ Beyond that, MALT1 is enhanced in patients with immunodeficiency‐polyendocrinopathy and immune‐dysregulation polyendocrinopathy enteropathy X‐linked syndrome.^18^ In the current study, MALT1 expression in AS patients was higher than that in healthy subjects. Possible explanations could be that (1) MALT1 induces immune dysregulation or T‐cell‐mediated autoimmunity via suppressing the differentiation of regulatory T cells into Th1 cells and Th17 cells, thus abnormally unregulated MALT1 could reveal a high risk of AS.^10^, ^19^ (2) MALT1 is excessively expressed and activated in osteoclasts, who are highly proliferated in AS pathology; therefore, MALT1 is consequently overexpressed in AS patients.^12^, ^13^

Some previous studies reveal that MALT1 maintains the homeostasis of CD4^+^ T cells and represses the differentiation of Th1 and Th17 cells in vitro and in vivo, whereas its correlation with Th cells in AS patients has never been explored.^9^, ^10^, ^11^ Our study found that MALT1 expression was positively correlated with Th17 cells in AS patients. One possible explanation for this could be that MALT1 interacts with caspase recruitment domain, B‐cell lymphoma 10, and subsequently mediates T‐cell regulation, to further enhance the differentiation of CD4^+^ T cells into Th17 cells.^8^ Besides, a preceding study investigates the correlation between MALT1 expression and clinical features in autoimmune diseases: MALT1 is positively related to CRP, TNF‐α, and IL‐17A in inflammatory bowel disease patients.^17^ In the current study, MALT1 expression was positively linked with IL‐17A, CRP, BASDAI, PGADA, and ASDAS_CRP_. Explanations for this could be that (1) MALT1 enhances NF‐κB signaling, whose activation is correlated with elevated inflammatory status in autoimmune diseases like AS; correspondingly, MALT1 overexpression is linked with enhanced inflammatory status in AS patients.^20^ (2) MALT1 positively links with Th1 and Th17 proportions and their secreted cytokines; therefore, elevated MALT1 might reveal an aggravated disease status; therefore, MALT1 expression is positively linked with AS activity.

However, previous studies never detect the association between MALT1 expression and treatment response of TNF inhibitor in AS patients. Interestingly, in the present study, MALT1 was decreased gradually in AS patients during the TNF inhibitor treatment; meanwhile, MALT1 was decreased after the treatment of TNF inhibitor in responding patients compared with non‐responding patients. Possible explanations could be that (1) MALT1 is positively linked with inflammation status; therefore, MALT1 expression is decreased as the inflammation status is alleviated by TNF inhibitor treatment. (2) Declining MALT1 represents the reduction in inflammation status, which is linked with the assessment of ASAS40; thus, MATL1 decrement along with treatment would reflect the response to TNF inhibitor in AS patients.^21^, ^22^

Besides, it would be interesting to discuss the reasons for stopping previous TNF inhibitors, which would be explained as follow: (1) TNF inhibitors could be discontinued after it achieves a certain efficacy. (2) TNF inhibitors might be discontinued for the heavy economic burden.

Some limitations still existed in the current study: (1) As a single‐center study, a patient‐selection bias might exist, which could affect the applicability of the study findings. (2) This study merely enrolled 73 AS patients; thus, the sample size was relatively small. (3) The current study merely evaluated the correlation of MALT1 expression with treatment efficacy of TNF inhibitor, but not with other biological agents such as IL‐17 antagonist; thus, the forthcoming study could explore this area.

Collectively, MALT1 positively correlates with Th17 cells, inflammatory, and activity degree; meanwhile, its decrement along with treatment reflects the response to TNF inhibitor in AS patients.

## CONFLICT OF INTEREST

The authors declare that they have no competing interests.

## Supporting information

Table S1

Figure S1

Figure S2

Figure S3

## Data Availability

The datasets used and/or analyzed during the current study are available from the corresponding author on reasonable request.
