# Supplementary material for: MALT1 positively relates to Th17 cells, inflammation/activity degree, and its decrement along with treatment reflects TNF inhibitor response in ankylosing spondylitis patients
Source: J Clin Lab Anal. 2022 May 27;36(7):e24472. doi: 10.1002/jcla.24472 (PMC9279967; doi:10.1002/jcla.24472)
Supplement: Supplementary file 1 — Table S1 [file JCLA-36-e24472-s002.docx]

**Supplementary table 1.** Association of MALT1 expression with Th17 cells, IL-17A, Th1 cells, IFN-γ, and Th1/Th17 ratio in the subgroups.

| Items | MALT1 expression | | | |
| --- | --- | --- | --- | --- |
|  | AS patients with ASAS40  response (n = 39) | | AS patients without ASAS40 response (n = 34) | |
|  | *r_s_* | *P* value | *r_s_* | *P* value |
| Th17 cells (%) | 0.421 | **0.041** | 0.211 | 0.372 |
| IL-17A (pg/mL) | 0.175 | 0.287 | 0.389 | **0.023** |
| Th1 cells (%) | 0.364 | 0.081 | 0.035 | 0.885 |
| IFN-γ (pg/mL) | 0.284 | 0.079 | 0.012 | 0.946 |
| Th1/Th17 ratio | -0.146 | 0.496 | -0.244 | 0.301 |

MALT1, Mucosa-associated lymphoid tissue lymphoma translocation protein 1; AS, ankylosing spondylitis; Th17, T helper 17; IL-17A, interleukin 17A; Th1, T helper 1; IFN-γ, interferon-gamma.
