# Supplementary figures and images for: MALT1 positively relates to Th17 cells, inflammation/activity degree, and its decrement along with treatment reflects TNF inhibitor response in ankylosing spondylitis patients
Source: J Clin Lab Anal. 2022 May 27;36(7):e24472. doi: 10.1002/jcla.24472 (PMC9279967; doi:10.1002/jcla.24472)

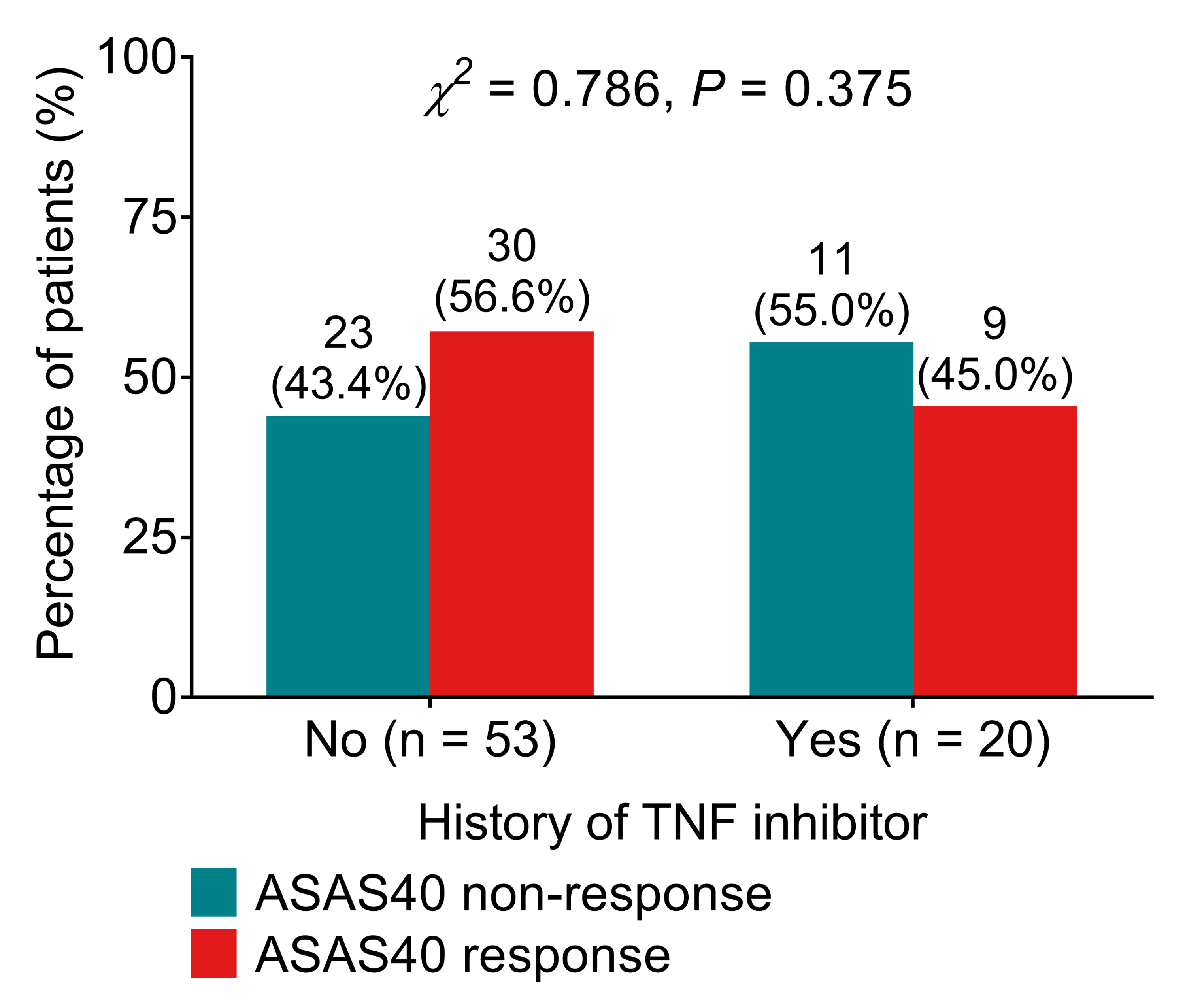

Supplement: Supplementary file 3 — Figure S2 [file JCLA-36-e24472-s003.tif]

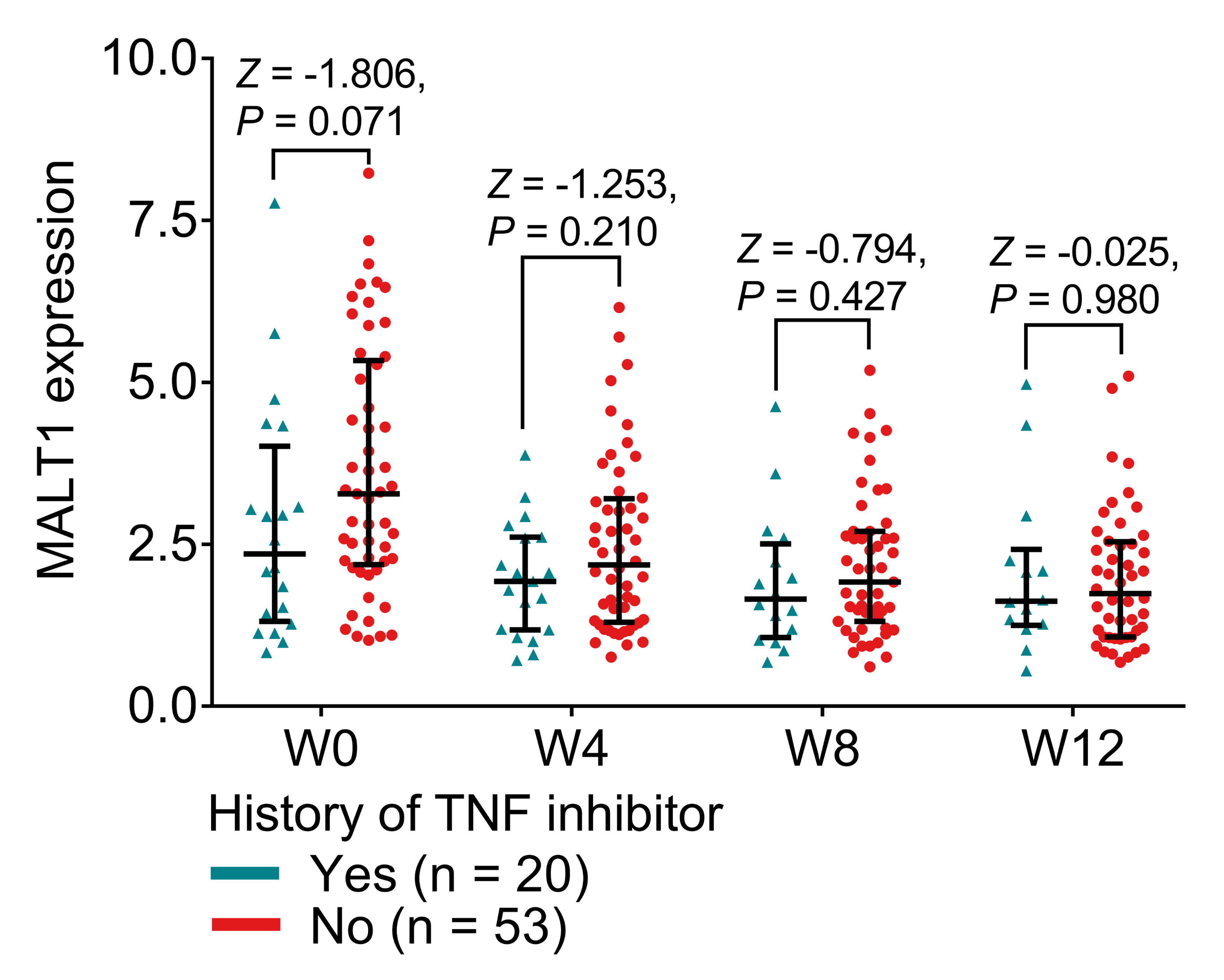

Supplement: Supplementary file 4 — Figure S3 [file JCLA-36-e24472-s004.tif]
